# Screening and brief intervention for alcohol and other drug use in primary care: associations between organizational climate and practice

**DOI:** 10.1186/1940-0640-8-4

**Published:** 2013-02-11

**Authors:** Erica Cruvinel, Kimber P Richter, Ronaldo Rocha Bastos, Telmo Mota Ronzani

**Affiliations:** 1 Federal University of Juiz de Fora -Rua José Lourenço Kelme, s/n University Campus-São Pedro, Rua São Mateus, 370, apto. 601, Bairro: São Mateus, CEP, 36025-000 Juiz de Fora, MG Brazil; 2 University of Kansas Medical Center, 3901 Rainbow Boulevard, 66160 Kansas City, KSUSA; 3 Federal University of Juiz de Fora - Rua José Lourenço Kelmer, s/nCampus Universitário, São Pedro, 36036-330 Juiz de Fora, MG Brazil; 4 Federal University of Juizde Fora - Rua José Lourenço Kelmer, s/n Campus Universitário, São Pedro, 36036-330 Juiz de Fora, MG Brazil

**Keywords:** Organizational climate, Screening, Brief intervention, Alcohol, Tobacco, Substance abuse

## Abstract

**Background:**

Numerous studies have demonstrated that positive organizational climates contribute to better work performance. Screening and brief intervention (SBI) for alcohol, tobacco, and other drug use has the potential to reach a broad population of hazardous drug users but has not yet been widely adopted in Brazil’s health care system. We surveyed 149 primary health care professionals in 30 clinics in Brazil who were trained to conduct SBI among their patients. We prospectively measured how often they delivered SBI to evaluate the association between organizational climate and adoption/performance of SBI.

**Methods:**

Organizational climate was measured by the 2009 *Organizational Climate Scale for Health Organizations,* a scale validated in Brazil that assesses leadership, professional development, team spirit, relationship with the community, safety, strategy, and remuneration. Performance of SBI was measured prospectively by weekly assessments during the three months following training. We also assessed self-reported SBI and self-efficacy for performing SBI at three months post-training. We used inferential statistics to depict and test for the significance of associations.

**Results:**

Teams with better organizational climates implemented SBI more frequently. Organizational climate factors most closely associated with SBI implementation included professional development and relationship with the community. The dimensions of leadership and remuneration were also significantly associated with SBI.

**Conclusions:**

Organizational climate may influence implementation of SBI and ultimately may affect the ability of organizations to identify and address drug use.

## Background

Several studies have demonstrated the effectiveness of screening and brief intervention (SBI) in reducing hazardous alcohol use. Implementing such tools in primary health care has the potential to help professionals identify and intervene on a very broad population and possibly reduce the prevalence of alcohol use [1–4] as well as tobacco use [5]. Although the efficacy for SBI for drug use is unknown, a strong argument can be made for its inclusion in protocols for alcohol and tobacco SBI [6].

One tool often used to screen for drug use is the Alcohol, Smoking and Substance Involvement Screening Test (ASSIST). The ASSIST is a simple screening instrument consisting of eight questions, which was developed by the World Health Organization (WHO) to identify patterns of consumption and problems related to alcohol, tobacco, and illicit drugs [7]. Screening tools may be paired with therapeutic brief interventions (BI) that focus on behavior change. Brief intervention is a technique, often based on motivational interviewing [8], that is delivered by caregivers to persons engaging in risky health behaviors. It is commonly delivered to persons who are not ready to change their behaviors. Brief interventions can last from 5 to 60 minutes and can be conducted in one to three sessions [9, 10].

Screening and brief intervention can be performed by professionals of varying backgrounds and educational levels. Previous studies suggest that the effectiveness of SBI for some drug use outcomes is identical, or even superior to, other interventions that require more time and more intensive training on the part of professionals [1, 9, 11].

Several countries are involved in an international multicenter project whose objective is to evaluate the implementation of SBI in primary health care (PHC). This model consists of detecting high-risk users of alcohol, tobacco, or other drugs using standardized screening tools and implementing BI in a single session [2, 12, 13]. In Brazil, PHC professionals have encountered numerous obstacles to incorporating SBI into their daily routine, including poor teamwork, low motivation, high turnover, high workload, lack of adequate infrastructure, bureaucracy, and poor organization of teamwork [3, 14].

It is possible that some of the obstacles related to the implementation of SBI are linked to the health-care work environment. Although prior studies in Brazil identified some potential environmental factors, no studies have systematically evaluated their impact on treatment practices. One of the ways commonly used to measure these factors is through the evaluation of organizational climate (OC). There is a consensus in the literature that OC influences the conduct and effectiveness of people who work within organizations [15]. Organizational climate can be defined as the formal or informal staff perceptions of policies, practices, and organizational procedures [15]. Studies in health services have found that organizational effectiveness depends on several characteristics of the context, including culture, structure, and OC [16–21].

It has been proposed that OC can influence the effectiveness of treatment, therapeutic alliance, treatment protocols, accountability, and continuity of services [22]. Several studies conducted within mental health service settings found OC to be related to various aspects of job satisfaction and turnover, which might be linked to quality of care and outcomes. Morris and Bloom [23] conducted a study among 148 administrators and staff of 17 community mental health centers in Colorado; aggregate perceptions about the climate and culture were strongly linked to job attitudes within each clinic. Aarons and Sawitzky [24] surveyed 301 staff at 49 mental health centers and found that positive attitudes toward the worksite were significantly associated with lower employee turnover, and there was a significant inverse relationship between OC and positive attitudes toward work. Glisson et al. [17], in their study of 100 mental-health clinic directors, found that OC explained a significant proportion of professional worker turnover. The turnover rate in clinics with a poor OC was two times higher than the rates in clinics with a positive OC. Despite the relevance of the topic, few studies specifically address OC and implementation of SBI for substance abuse, although one study among 95 primary-care health professionals in Brazil found an association between the use of participatory decision-making within health-care teams and implementation of SBI [25].

The purpose of this study was to evaluate perceived OC among primary health care providers in Brazil who participated in SBI training and to prospectively assess their SBI behaviors to better describe the effects of work environment on SBI implementation. The study has strong potential for improving understanding of how SBI might be adopted in Brazil. Brazil has a universal health care system based on primary health-care teams that operate under very similar resource and administrative structures throughout the country.

## Methods

### Participants

The study was conducted in a convenience sample of four towns with populations ranging from 8791 to 154,547 in the states of Rio de Janeiro and Minas Gerais Brazil. Cities had to meet the following conditions to participate: 1) have at least one PHC unit in the town; 2) provide a letter of approval from the local Department of Health; 3) make all PHC teams available to participate in the study; 4) ensure the availability of all PHC teams for classroom training and follow-up.

Professionals had to meet the following criteria to participate in training and to have their OC surveys included in study analyses: 1) participation in modules 1 and 2 of classroom SBI training; 2) provision of written informed consent; 3) continued employment at the same PHC throughout the course of the study; and 4) completion of all survey assessments. Professionals signed an attendance sheet to verify training participation. The study was approved by the Ethics Committee of the Federal University of Juiz de Fora.

### Design

This study was part of a larger project entitled "Process Evaluation of Prevention Practices related to Drug Abuse and Domestic Violence in Primary Health Care Services," which was developed by the University’s Center of Research in Social Psychology and Health (POPSS /UFJF). For the study described herein, we secured municipal approval, provided city-wide seminars to raise awareness of problems related to drug abuse, provided SBI training, provided ongoing on-site technical assistance and SBI assessment of PHC teams, and conducted follow-up assessments of OC and SBI. To secure approval for the project, we first contacted Brazil’s Family Health Program, which over sees PHC in each city and is housed within the Department of Health. We worked with the Family Health Program to develop feasible study procedures and secure approval from the Departments of Health in each participating city. Following approval, we held seminars within each city to raise awareness about the topic. All PHC clinicians and professionals of the municipalities (including administrative staff) were invited to attend the seminars. In addition, professionals from mental health centers; representatives of the Municipal Health Council; and representatives of Alcoholics Anonymous, the Military Police, the Protection and Advice Council on Drugs, hospitals, and religious institutions were invited to participate in the seminars.

Each seminar addressed the problems of drug abuse, its prevalence, and methods for early detection and brief intervention. Afterwards, the project design and proposal for implementation in each municipality were presented. During the seminar, the PHC teams that showed interest in the project were invited to participate. One of the selection criteria was the availability for training of the entire team.

The third step included training and follow-up. All clinicians and professionals of participating PHC clinics (including administrative staff and community health workers) were invited to attend. Community health workers are persons with a high school diploma or technical school training who usually live in the community served by the clinic for which they work. They have no formal training in clinical health care procedures. They are skilled in community-based health promotion, including providing in-home outreach services and case identification. They serve as a link between the PHC clinics and the community.

We developed a standardized eight-hour classroom training covering the following topics: basic concepts and facts about alcohol and other drug abuse and its consequences for public health; use of screening instruments (e.g., the ASSIST); use of brief intervention for different levels and types of drug use; implementing SBI in clinical practice; and the role of teamwork. Brief intervention strategies discussed in the training were based on the principles of motivational interviewing, using the Feedback, Responsibility, Advice, Menu of options, Empathy, and Self-efficacy (FRAMES) counseling approach. We provided examples of how to address each of these components for different types of drugs of abuse and provided opportunities for participants to practice with each other based on treatment scenarios provided by study staff.

Following the classroom training, we continued ongoing training and monitoring of the PHC teams for three months. Research staff provided PHC teams with ASSIST forms that included an additional question asking if the provider delivered a brief intervention (BI) to each patient screened. The form directed clinicians to complete the ASSIST for all patients and included space to report any type of BI that clinicians provided to any patient. Brief intervention was defined as providing guidelines-based information about risky drinking to low-risk and abstinent patients as well as providing FRAMES-based intervention to moderate or high risk users.

At weekly meetings, study staff collected the ASSIST screening forms and summarized the data into a clinic log. The log included the name of the city, the PHC team, the name of the professional, the number of ASSIST screenings and BIs conducted, and the date when each instrument was applied. Research staff provided feedback on the prior week’s ASSIST and BI performance and led an interactive discussion on how to improve performance in the clinic. Research staff graphed ASSIST and BI performance by each clinic and professional, on to standardized feedback forms that included: screening date, number of ASSIST screens applied, and whether the professional completed BI (yes/no). At the clinic session, these feedback forms were provided as handouts to clinic staff. Research staff described clinic performance, then asked open-ended questions about SBI practice. These questions were used to prompt an interactive discussion on how to improve performance the next week. After three months, trained research staff administered the Organizational Climate Scale (described below) along with a self-report scale on SBI performance and self-efficacy to participating clinicians on site.

### Instruments

The Organizational Climate Scale used in this study was developed and validated to measure the perceptions of health care professionals [26]. It consists of 64 items with Likert-scale responses (1=strongly disagree, 2=slightly disagree, 3=neither agree nor disagree, 4=slightly agree, 5=strongly agree). Items are grouped into the following seven domains with good reliability, as measured by Cronbach's alpha: 1) leadership (α=0.872); 2) professional development (α=0.934); 3) relationship and team spirit (α=0.837); 4) relationship with the community (α=0.839); 5) safety (related to work procedures and the overall workplace, α=0.772); 6) strategy (participatory decision-making regarding team strategies to address issues, α=0.812); and 7) remuneration (α=0.834).

We assessed SBI practices and self-efficacy using self-administered surveys of participants. This questionnaire included four practice-related questions specifically addressing how often professionals carried out activities to prevent risky use of alcohol. Practice 1 measured the frequency with which the professional asked patients about their alcohol use. Practice 2 measured the frequency with which the professional provided BI to patients who screened positive for risky alcohol consumption. (Practice 1 and Practice 2 included the following response options: 1=every time, 2=the most of time, 3=sometimes, 4=rarely or never, 5=not part of my job.) Practice 3 measured professionals’ readiness to intervene with drug users. (This question had four response options: 1=I am not considering intervening; 2=I'm thinking about intervening, but have not yet started; 3=I intervene sometimes; 4=Intervening is already part of my work routine.) Finally, Practice 4 measured provision of preventive interventions, including providing drug use education, such as lectures or others educational interventions in PHC patient groups or at community health fairs. (This component had five response options: 0=never, 1=two to three times a year, 2=monthly, 3=weekly, 4=every day.)

The survey also included two scales. The first assessed perceived self-efficacy for performing SBI, with four questions that included Likert-type response options (1=strongly disagree, 2=disagree, 3=neither agree nor disagree, 4=agree, 5=strongly agree). The second measured confidence for developing SBI activities, consisting of 10 items with Likert-type response options (1=not confident, 2=very little confidence, 3=moderate degree of confidence, 4=very confident [27]. Some of the factors assessed in this scale included confidence in screening for alcohol problems using the CAGE, AUDIT, or ASSIST; confidence in referring patients for further diagnostic assessment or treatment; and confidence in providing advice regarding low-risk alcohol consumption. The reliability of the confidence scale was α=0.91, and the reliability of the self-efficacy scale was α=0.76; both are satisfactory, as Cronbach’s α values above 0.70 are considered to have a good level of internal consistency. A sociodemographic questionnaire was used to collect personal and professional characteristics including age, gender, education, position, and time of service. This instrument was administered just prior to classroom training.

### Data analysis

Data were entered into IBM® SPSS version 15.0 statistical software. To estimate our data entry error rate, we selected a random sample of 30% of questionnaires and compared the data entered with the paper versions. The error rate was lower than 5%, so we proceeded to analysis of the electronic data. To ensure data quality, researchers with advanced training in data management implemented a system to ensure valid and accurate data entry, including methods to avoid missing and out-of-range values. The data managers reviewed data before entering it and examined entered data for missing and out-of-range values.We aggregated data by clinic teams to control for shared OC within each team. We then tested for the significance of these associations using inferential statistics.

Each team consists of physicians, nurses, community health workers, and other health professionals. We believe the team, rather than the facility, is most responsible for the perceived OC for each worker. Each team operates as an independent administrative unit with separate leadership. Thus, the weighted average on the scales and the practical issues of the 149 subjects were grouped into 30 PHC teams, with the team as the unit of analysis. Although aggregating data by teams reduced our power to detect associations, it was important to do so to account for the clustering of data due to the shared work environment.

We assessed whether the distribution of scores on the scales were normal using the Kolmogorov-Smirnov test. This permitted the use of parametric tests for inferential analyses. We then examined correlations between the subscales of OC, the number of ASSIST screens and BIs performed, and self-reported SBI practices, confidence, and self-efficacy. Correlation analysis was performed by the average aggregate values per team. To test for the significance of these correlations, we used the Pearson chi-square test with a cut off of p<0.05 for rejecting the null hypothesis that higher scores on the OC subscales related to lower rates of SBI or lower scores in OC related to higher rates of SBI.

### Ethical approval

This project was approved by the Ethics Committee at the Federal University of Juiz de Fora. All who agreed to participate signed a consent form that provided detailed information on the study, stated that participation was voluntary, noted that no compensation would be provided, included a confidentiality and privacy statement, and assured potential participants that no harm would occur should the provider choose not to participate.

## Results

### Study participants

The four municipalities included in this study had a total of 30 PHC teams. Two hundred and thirty professionals completed the theoretical training. The final survey sample consisted of 149 professionals, of whom 70.6% were community health workers, 6.6% were nurses, 5.9% were nursing assistants, 2.9% were physicians, 1.5% were social workers, and 12.5% were other professionals (including dentists, dental assistants, psychologists, and physical education teachers). The 81 professionals who completed the training but whose data were not included in analyses of OC were either no longer employed in the PHC unit or had been reassigned to another team.

### Correlation between OC and SBI practices and attitudes

Table 1 depicts correlations in data aggregated by team between SBI performance, self-reported SBI, and confidence/self-efficacy to perform SBI. There were a number of significant correlations between OC factors and clinic practice. The factors professional development and relationship with the community demonstrated the highest number of correlations with team practices and attitudes related to SBI. These two factors also demonstrated the strongest correlations with SBI practice. Likewise, leadership and remuneration had correlation coefficients higher than 0.40. Conversely, team spirit and strategy showed few significant correlations. Workplace safety had no significant correlations with any of the issues assessed. All significant correlations were positive, showing an association in the same direction of the associated variables; that is, the better the perception of OC, the higher the SBI practice and attitude scores.

**Table 1:** **Correlation between organizational climate and screening and brief intervention practices and attitudes (N = 30 Teams)**

	Questions about the practice of drug-use prevention activities							
OC scale factors	Practice 1	Practice 2	Practice 3	Practice 4	Implementation of ASSIST	BIs performed	CNF SBI	SE SBI
Leadership	0.307	0.423*	0.465**	0.346	0.286	0.333	0.345	0.460*
Professional development	0.455*	0.505*	0.499**	0.412*	0.548**	0.498**	0.339	0.294
Team spirit	−0.004	0.364*	0.308	0.326	0.182	0.111	0.031	0.244
Relationship with community	0.461*	0.616**	0.417*	0.106	0.232	0.376**	0.452*	0.366
Strategy	0.433*	0.378*	0.324	0.347	0.334	0.220	0.087	0.175
Remuneration	0.454*	0.467*	0.242	0.088	0.332	0.286	0.451*	0.209
Safety	0.312	0.231	0.311	0.065	0.322	0.179	−0.031	−0.225

## Discussion

Our findings suggest that positive organizational climate is associated with enhanced performance of SBI for alcohol and substance abuse problems. Across 30 primary health care clinics in Brazil, multiple domains of OC were directly associated with self-reported use of SBI. Of seven OC factors, six showed significant positive correlations with SBI. In addition, several OC factors were also directly related to confidence and self-efficacy to perform SBI.

Before discussing the results in detail, it is important to consider a design limitation of the study and the degree to which it affects interpretation of the findings. Although it appears that OC influenced adoption of SBI, we measured OC at the end, rather than the beginning, of the study. It may be that our SBI training also affected OC within those teams, in which case we may be reporting an association that is not a causal relationship. Because we did not measure OC prior to measuring SBI performance, and we did not have a control group, our design does not rule out this possibility. However, we believe this is unlikely. Our classroom training was very focused on the concepts of SBI and how to deliver it in clinics; hence, we do not believe that aspect of the intervention could have strongly affected OC. It is possible our weekly performance feedback sessions could have affected OC somewhat, as these meetings involved interactive discussions of how to incorporate SBI into clinic practice, and at times may have touched on leadership, teamwork, and/or relationships with community members. However, even after the SBI training and feedback, clinics differed widely in their perceived OC, suggesting that the intervention effects on OC, if they occurred, were weak. Hence, we believe the OC values we observed at post training are likely to be similar to those we would have observed at baseline, as only three months elapsed between baseline and follow-up assessment.

Even after training and feedback in all participating clinics, there was some variation in adoption of SBI, and this variation may be due to underlying clinic-specific OC factors. Hence, OC may moderate the impact of training on the adoption of new clinical practices such as SBI. Some authors have found that in environments with a favorable OC, activities become more easy to perform, produce greater job satisfaction, and help workers reach their full potential [17, 28]. Clinicians who work in environments with less rigidity and more functional climates report higher levels of organizational commitment [17], which is important for incorporating new activities, such as SBI, into work routines.

To better understand and test this hypothesis, it would be useful to include other measures of OC in future trials to ensure all domains are included; i.e., assess OC at the beginning of a controlled trial of SBI training in clinics; include clinics with a wide range of OC in both study arms; and perhaps even design the study to explore the impact of directly addressing OC during SBI training. The analytic plan of such a trial could explicitly plan for analyses that examine the moderating effects of baseline OC on SBI performance. To better understand how specific domains of OC might moderate the effects of SBI training, we further explore the findings of the present study below.

Professional development (an OC factor) was most closely linked to SBI performance. The 11 items comprising this factor assess learning via trainings offered in the unit, improvement in productivity, investment in the personal and technical development of staff, application of newly acquired skills, and other features of environment supportive of development. Our findings accord with numerous studies that have demonstrated that training increases knowledge, builds confidence in addressing clinical topics [27, 29], and helps prevent burnout [30].

The second factor most frequently associated with SBI was relationship with the community. This factor assesses the strength of relationships within the health teams, between the teams and their communities, and between the teams and the municipal health department. A strong relationship with community residents is vital to the mission of Brazilian primary care because of the daily presence of PHC teams in the community and their mission to make preventive care accessible to all. Teams with strong community relationships may be more likely to implement SBI because they have caring and trusting relationships with families, which enables them to address sensitive issues such as drug problems. Studies show that community health workers (who made up the majority of our study sample) vary in terms of their implementation of drug prevention and treatment activities [27]. Such workers, however, are key in bringing together the community and health services. They have strong potential for developing SBI interventions and for involving families, schools, and philanthropy organizations in early intervention activities [29].

Leadership was also associated with several measures of SBI implementation. The 17 items comprising this factor include supervision as well as motivation—two important aspects of leadership (e.g., “*my manager is able to motivate my team, my manager corrects errors as they occur, my manager rewards good work, communication with my manager is clear and open, my manager keeps informed regarding the goals of the team”*). Our findings support the suggestion by Patterson et al. [20] that clinical supervision is important to producing better services and patient outcomes. They argue it is important because supervision improves OC (and hence performance), supports implementation and quality of evidence-based practice, increases the visibility of professional counseling, and improves patient outcomes.

Remuneration was also related to SBI. This supports numerous studies that have linked remuneration, and perceived remuneration, with work performance and burnout [31, 32]. A study conducted among mental health professionals showed that the perceived balance between the effort to perform tasks and the reward was directly related to performance of evidence-based practice [31]. Another study conducted among mental health workers found the perception of high reward was a protective factor for risk of burnout [32].

The factors least associated with SBI practices were strategy and team spirit. This result was different from the findings of several studies of OC that support the importance of teamwork forperformance and quality of services [33, 34]. It is important to highlight that remuneration and strategy were associated with some attitudes for substance use prevention but showed no relationship with SBI measures. Many professionals develop approaches to reducing the consumption of alcohol or other drugs, without necessarily using structured and standardized instruments such as the ASSIST. Similarly, some PHC workers could provide advice for behavior change even when they do not follow all the recommended steps in evidence-based BI.

In general, although self-reported SBI practices related to a number of OC factors, in terms of objectively measured behaviors, only professional development and relationship with community were significantly related to performance of SBI. The other OC dimensions of leadership, relationship, team spirit, worksite safety, team strategy, and remuneration did not correlate with SBI performance. Perhaps only teams that valued and participated in professional development were motivated to adopt new interventions such as SBI. Also, teams with strong links to the community might be more aware of the impact of drug use on their communities, less willing to judge and/or condemn patients with drug problems, and more willing to adopt new interventions that could benefit their communities, such as SBI.

Interestingly, professional development did not relate to confidence or self-efficacy to perform SBI, and relationship with community only related to confidence to perform SBI. Professional development activities for our participants probably had not ever addressed the treatment of drug abuse, because it has never been a mandate to address it in primary health care in Brazil. Perhaps having a strong relationship with the community would enable professionals to overcome reservations about addressing a sensitive and often illegal behavior, for the good of their patients.

This study had several limitations. First, this paper relates perceived OC with performance of BI. However, many more administrators and higher level clinic staff participated in the intervention than were included in the final OC survey. Our data on OC is based on 149 participants for whom we had complete survey data. These were mainly community health workers. However, a total of 230 health care providers completed the classroom part of the training, and most health care providers and administrators in participating clinics attended the post-training weekly clinic meetings in which we assessed ASSIST and BI performance and provided feedback. Hence, a much greater proportion of providers and administrators were probably exposed to the training and site intervention compared with the number who completed the OC assessment. The differences between the intervention population and the survey population may have reduced how representative the survey results were, may have introduced bias into the findings, and limited the number and type of analyses we were able to perform.

Second, given the nature of the data, it would have been better to use an analysis framework, such as mixed effects regression, that directly addressed the clustering and hierarchy. The sample in this study included 149 professionals distributed in 30 teams, including teams with less than five individuals observed in four municipalities. Although the method of aggregation we used was simpler and involved fewer assumptions, some statistical power may have been lost compared to a true multilevel analysis. Future studies should include greater representation within teams and examine whether clustering and hierarchy have effects on relationships between OC and SBI performance.

Third, our prospective measures of SBI performance relied on health-care provider administration of the ASSIST. It is possible that providers misreported screening rates to comply with the research protocol. We believe this is unlikely, as it would be difficult and time-consuming to fill out multiple ASSIST forms for nonexistent patients given that staff are extremely busy, and because we saw a great deal of variance in SBI performance across and within teams.

Fourth, factors other than OC may affect performance of SBI. Future studies could relate OC with infrastructure or other pre- or co-occurring conditions that could affect SBI performance, such as private versus public health care contexts, resources available to clinics, or specialist versus primary health care providers.

Last, although all of our participants attended the same SBI training, we do not know what other professional development activities they have attended or the extent to which their clinics supported ongoing professional development. Futures studies should assess the extent to which ongoing professional development opportunities contribute to receptiveness and ability to innovate among providers.

## Conclusions

In general, the findings suggest that positive OC is related to greater adoption of SBI for alcohol and other drug use. Organizational climate may influence implementation of SBI and ultimately may affect the ability of organizations to identify and address drug use. Future research could identify effective culture change processes, incorporate them into SBI trainings, and examine whether changes in OC facilitate implementation. In this way, SBI implementation could facilitate broader changes that could be generalized to other areas of health care delivery.
